# Risk Factors for Reinfection with SARS-CoV-2 Omicron Variant among Previously Infected Frontline Workers

**DOI:** 10.3201/eid2903.221314

**Published:** 2023-03

**Authors:** Katherine D. Ellingson, James Hollister, Cynthia J. Porter, Sana M. Khan, Leora R. Feldstein, Allison L. Naleway, Manjusha Gaglani, Alberto J. Caban-Martinez, Harmony L. Tyner, Ashley A. Lowe, Lauren E.W. Olsho, Jennifer Meece, Sarang K. Yoon, Josephine Mak, Jennifer L. Kuntz, Natasha Schaefer Solle, Karley Respet, Zoe Baccam, Meredith G. Wesley, Matthew S. Thiese, Young M. Yoo, Marilyn J. Odean, Flavia N. Miiro, Steve L. Pickett, Andrew L. Phillips, Lauren Grant, James K. Romine, Meghan K. Herring, Kurt T. Hegmann, Julie Mayo Lamberte, Brian Sokol, Krystal S. Jovel, Mark G. Thompson, Patrick Rivers, Tamara Pilishvili, Karen Lutrick, Jefferey L. Burgess, Claire M. Midgley, Ashley L. Fowlkes

**Affiliations:** University of Arizona, Tucson, Arizona, USA (K.D. Ellingson, J. Hollister, C.J. Porter, S.M. Khan, A.A. Lowe, Z. Baccam, F.N. Miiro, J.K. Romine, K.S. Jovel, P. Rivers, K. Lutrick, J.L. Burgess);; Centers for Disease Control and Prevention, Atlanta, Georgia, USA (L.R. Feldstein, J. Mak, Y.M. Yoo, J. Mayo Lamberte, M.G. Thompson, T. Pilishvili, C.M. Midgley, A.L. Fowlkes);; Kaiser Permanente Northwest, Portland, Oregon, USA (A.L. Naleway, J.L. Kuntz); B; aylor Scott and White Health, Temple, Texas, USA (M. Gaglani); A.J. Caban-Martinez, N. Schaefer Solle);; St. Luke’s Regional Health Care System, Duluth, Minnesota, USA (H.L. Tyner, K. Respet, M.J. Odean);; Abt Associates Inc., Rockville, Maryland, USA (L.E.W. Olsho, M.G. Wesley, S.L. Pickett, M.K. Herring, B. Sokol);; Marshfield Clinic, Marshfield, Wisconsin, USA (J. Meece);; University of Utah Health, Salt Lake City, Utah, USA (S.K. Yoon, M.S. Thiese, A.L. Phillips, K.T. Hegmann)

**Keywords:** SARS-CoV-2, omicron variant, COVID-19, respiratory infection, viruses, coronaviruses, vaccine effectiveness, mRNA vaccines, reinfection, risk factors, frontline workers, zoonoses, United States

## Abstract

In a cohort of essential workers in the United States previously infected with SARS-CoV-2, risk factors for reinfection included being unvaccinated, infrequent mask use, time since first infection, and being non-Hispanic Black. Protecting workers from reinfection requires a multipronged approach including up-to-date vaccination, mask use as recommended, and reduction in underlying health disparities.

Essential and frontline workers experience repeated occupational exposure to SARS-CoV-2 (*1*). The variant B.1.1.529 (Omicron) is characterized by unprecedented transmissibility and potential for immune evasion, which led to a surge in first-time infections and reinfections in the United States beginning in December 2021 (*2*). Before Omicron predominance, previous infection had been highly protective against reinfection, but protection waned as Omicron emerged (*3**–**5*). Compared with results for pre-Omicron variants, reports of mRNA vaccine effectiveness against first-time Omicron infections were lower (46% after 2 doses and 60% after 3 doses) (*6*). Early reports on vaccine effectiveness against hospitalization related to reinfection with Omicron demonstrated 35% risk reduction with 2 doses of mRNA vaccine and 68% with 3 doses (*6*,*7*).

Little is known about the constellation factors that put persons who had previous infections at risk for reinfection with Omicron (*8*). In this study, we examined a prospective cohort of essential and frontline workers who had previous SARS-CoV-2 infections to identify risk factors for reinfection during Omicron predominance.

## The Study

Beginning in July 2020, frontline workers in 8 US locations were enrolled in the Arizona Healthcare, Emergency Response, and Other Essential Workers Study (AZ-HEROES) and the Research on the Epidemiology of SARS-CoV-2 in Essential Response Personnel (RECOVER) Study (*9*,*10*). Participants self-collected weekly mid-turbinate nasal specimens regardless of symptoms and upon the date of onset of any COVID-19–like illness symptoms. All specimens were tested at Marshfield Clinical Research Laboratories (Marshfield, WI, USA) for SARS-CoV-2 RNA by reverse transcription PCR (RT-PCR) testing. Specimens positive by RT-PCR with a cycle threshold (Ct) value <30 underwent whole-genome sequencing to determine SARS-CoV-2 variant. (Ct is defined as the number of cycles required for the fluorescent signal to cross the threshold [i.e., exceeds background level].) 

If the specimen was ineligible (Ct >30) for sequencing, the date of incident infection was used to estimate variant predominance by using the state-specific date at which >50% of specimens sequenced were of the Delta or Omicron variants according to Centers for Disease Control and Prevention data (Figure) (*11*). Only participants who were actively enrolled in the study during site-specific Omicron predominance, and those who had 1 previous infection at least 45 days before Omicron predominance, were included (Figure).

**Figure:**
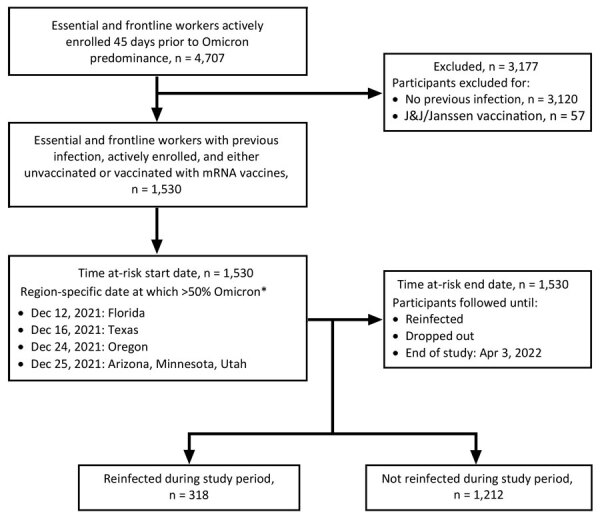
Sample inclusion criteria and site-specific study timeline for risk factor analyses for reinfection with SARS-CoV-2 Omicron variant among previously infected frontline workers, United States.

We identified first infections on the basis of participant-reported positive viral test results before enrollment or positive study-based RT-PCR results after enrollment. We defined reinfections as the second incident infection with >90 days between last date of positivity for first infection and first date of positivity for the second infection, or a second infection after 45 days from a distinct SARS-CoV-2 variant. We collected demographic and underlying health information at enrollment. Participants self-reported vaccination at enrollment, quarterly, and more frequently when new vaccines became available; study staff subsequently verified self-reports through vaccine card uploads, state vaccine registries or electronic medical records. We collected exposure variables, including self-reported mask use and number of hours worked per week, monthly and averaged them over the study period (*12*). All participants provided written consent, and protocols were approved by institutional review boards at all participating sites, including Abt Associates and the Centers for Disease Control and Prevention.

We estimated unadjusted and adjusted hazard ratios for Omicron reinfection with Cox proportional-hazards models by using the Andersen–Gill extension to account for time-varying vaccination status described previously (*13*). Adjusted models included demographic information, preexisting health conditions, time-varying vaccination status, self-reported mask use in the community, and time since previous infection. All statistical analyses were conducted in R version 4.0.4 (The R Project for Statistical Computing, https://www.r-project.org) and SAS university edition 9.4 (SAS Institute, Inc., https://www.sas.com).

Of 4,707 actively enrolled HEROES-RECOVER participants, 1,587 (33.7%) had a previous SARS-CoV-2 infection >45 days before Omicron predominance. We excluded from the study population persons who had received J&J/Janssen (https://www.jnj.com) vaccination (n = 57) (Figure). From the location-specific date of Omicron predominance through April 3, 2022, a total of 1,530 participants who had previous SARS-CoV-2 infections contributed 124,665 person-days at risk for reinfection (Table). More than half of the participants were from Arizona (59.7%), female (60.5%), and non-Hispanic White (66.9%). In this study sample, 42.5% were healthcare personnel, 26.1% first responders, and 31.4% other essential workers.

**Table:** Composition of study sample at risk for reinfection with SARS-CoV-2 during Omicron predominance, United States, December 2021‒April 2022*

Characteristic	No. participants, n = 1,530†	No. reinfected, n = 318	No. person-days at risk, n = 124,665	Reinfection incidence/1,000 days at risk (95% CI)	Unadjusted HR (95% CI)‡	Adjusted HR (95% CI)§
Site, no. (%)						
Tucson, AZ	579 (37.8)	109	47,933	2.27 (1.85‒2.70)	Referent	Referent
Phoenix, AZ	211 (13.8)	40	17,054	2.35 (1.62‒3.07)	1.03 (0.72‒1.49)	0.99 (0.66‒1.48)
Other areas in AZ	123 (8.0)	21	10,389	2.02 (1.16‒2.89)	0.88 (0.56‒1.42)	0.75 (0.46‒1.35)
Temple, TX	93 (6.1)	20	8,003	2.50 (1.40‒3.59)	1.15 (0.71‒1.85)	1.12 (0.68‒1.82)
Portland, OR	59 (3.9)	9	4,851	1.86 (0.64‒3.07)	0.84 (0.42‒1.65)	0.99 (0.48‒2.01)
Miami, FL	151 (9.9)	43	11,834	3.63 (2.55‒4.72)	1.66 (1.17‒2.37)	1.28 (0.86‒1.91)
Duluth, MN	135 (8.8)	24	11,271	2.13 (1.28‒2.98)	0.92 (0.59‒1.43)	1.08 (0.67‒1.74)
Salt Lake City, UT	179 (11.7)	52	13,330	3.90 (2.84‒4.96)	1.65 (1.19‒2.30)	1.61 (1.09‒ 2.37)
Age category, y, no. (%)						
18–34	360 (23.5)	83	28,112	2.95 (2.32‒3.59)	Referent	Referent
35–49	662 (43.3)	135	54,065	2.50 (2.08‒2.92)	0.86 (0.66‒1.13)	0.88 (0.65‒1.20)
50–77	508 (33.2)	100	42,488	2.35 (1.89‒2.81)	0.82 (0.61‒1.10)	0.89 (0.63‒1.27)
Sex, no. (%)						
M	605 (39.5)	144	47,929	3.00 (2.51‒3.50)	Referent	Referent
F	925 (60.5)	174	76,736	2.27 (1.93‒2.60)	0.76 (0.61‒0.95)	0.95 (0.72‒1.25)
Race/ethnicity, no. (%)¶#						
Non-Hispanic/White	1,023 (66.9)	197	84,350	2.34 (2.01‒2.66)	Referent	Referent
Non-Hispanic/Black	44 (2.9)	15	3,058	4.91 (2.42‒7.39)	2.02 (1.19‒3.41)	2.14 (1.17‒3.92)
Non-Hispanic/Asian	30 (2.0)	5	2,636	1.90 (0.23‒3.56)	0.81 (0.33‒1.96)	1.02 (0.43‒2.43)
Hispanic	375 (24.9)	88	29,837	2.95 (2.33‒3.57)	1.26 (0.98‒1.62)	1.30 (0.98‒1.72)
Other	18 (1.2)	3	1,605	1.87 (0.00‒3.98)	0.81 (0.26‒2.56)	0.56 (0.18‒1.78)
Comorbid conditions, no. (%)#**						
0	970 (63.4)	206	78,492	2.62 (2.27‒2.98)	Referent	Referent
>1	489 (32.0)	106	39,704	2.67 (2.16‒3.18)	1.02 (0.80‒1.29)	1.17 (0.90‒1.53)
Occupation, no. (%)††						
Heathcare personnel	650 (42.5)	126	53,745	2.34 (1.94‒2.75)	Referent	Referent
First responder	400 (26.1)	111	30,557	3.63 (2.96‒4.31)	1.52 (1.18‒1.96)	1.13 (0.78‒1.63)
Other essential worker	480 (31.4)	81	40,363	2.01 (1.57‒2.44)	0.86 (0.65‒1.13)	0.82 (0.60‒1.13)
Time-varying vaccination status, no.‡‡						
0 doses	441	125	32,879	3.80 (3.14‒4.47)	Referent	Referent
1 dose	34	5	2,605	1.92 (0.24‒3.60)	0.56 (0.22‒1.40)	0.56 (0.22‒1.42)
2 doses	646	107	48,039	2.23 (1.81‒2.65)	0.58 (0.45‒0.75)	0.57 (0.43‒0.75)
3 doses	513	81	42,726	1.90 (1.48‒2.31)	0.55 (0.41‒0.72)	0.54 (0.39‒0.75)
Mask use in community, no. (%)#§§						
Above mean (47%)	766 (50.1)	126	64,630	1.95 (1.61‒2.29)	Referent	Referent
Below mean	735 (48.0)	189	57,595	3.28 (2.81‒3.75)	1.64 (1.31‒2.06)	1.39 (1.07‒1.82)
Weekly work hours, no. (%)#						
Below mean (30 hours)	781 (51.0)	154	64,077	2.40 (2.02‒2.78)	Referent	Referent
Above mean	741 (48.5)	164	59,901	2.74 (2.32‒3.16)	1.14 (0.91‒1.41)	1.09 (0.87‒1.37)
Time elapsed since first infection, y, no. (%)						
<1	781 (51.0)	133	65,239	2.04 (1.69‒2.39)	Referent	Referent
>1	749 (49.0)	185	59,426	3.13 (2.66‒3.56)	1.53 (1.23‒1.91)	1.63 (1.28‒2.07)

*HR, hazard ratio. †Column percentage reported. ‡Each covariate listed was entered into a Cox proportional hazard model as a single predictor to generate unadjusted hazard ratios §All covariates were entered into a fully adjusted model. ¶Other includes American Indian or Alaska Native, Asian/Pacific Islander, or multiracial. #Missing values for this variable but <5% of study sample; persons who had missing values were excluded from hazard ratio analyses. **Includes asthma, other chronic lung disease, cancer, diabetes, heart disease or condition, hypertension, immunosuppression, kidney disease, liver disease, neurologic disease or disorder, autoimmune disease, or other medical problem requiring clinical care for >6 months. ††Includes inpatient, ambulatory, and institutional healthcare personnel; First responders include nonfire emergency medical service workers, fire services, law enforcement and corrections personnel; other essential workers include persons who work in the hospitality, retail, food service, education, government, or grocery sectors and persons who work in essential infrastructure and operations.  ‡‡Vaccination status was allowed to vary over the time period at risk by using the Andersen–Gill extension methods; column percentages are not provided because they do not add up to 100%.  §§Refers to the reported percentage of time masks were worn while in public but not at work in the past 7 days averaged across the study period.

Of all mRNA vaccines received, 71.2% were BNT162b2 (Pfizer-BioNTech, https://www.pfizer.com) and 28.8% were mRNA-1273 (Moderna, https://www.modernatx.com). About half (51.0%) of participants experienced their first infection within a year of location-specific Omicron predominance. Reinfections were identified among 318 (20.8%) participants; 27.0% of those reinfected were asymptomatic.

The proportional hazards assumption was met for all models. Participants who had 2 or 3 doses of mRNA vaccine had lower risk for reinfection with Omicron than persons who were unvaccinated (adjusted HR [aHR] 0.57 [95% CI 0.43–0.75] for those with 2 doses; aHR 0.54 [95% CI 0.39–0.75] for those with 3 doses) (Table). Although age and sex did not significantly predict reinfection in this sample, residence in Utah (aHR 1.61, 95% CI 1.09–2.37; referent Tucson, AZ, USA) and self-identification as non-Hispanic Black (aHR  2.14, 95% CI 1.17–3.92; referent non-Hispanic White) were risk factors for reinfection. Participants who wore masks in community settings less frequently, defined as wearing less than the mean reported percentage of time of 47% (interquartile range 8%–83%), had higher risk for reinfection (aHR 1.39, 95% CI 1.07–1.82). Finally, participants for whom >1 year had elapsed since their first infection had increased risk for reinfection compared with persons who had <1 year since their first infection (aHR 1.63, 95% CI 1.28–2.07).

## Conclusions

In this prospective cohort of previously infected frontline workers, mRNA vaccination with 2 or 3 doses reduced the risk for reinfection by >40%. Risk for reinfection was increased by low self-reported mask use and for persons who had an initial infection >1 year before the study period. Those findings are consistent with risk factor studies for primary infections and suggest that infection-induced immunity wanes over time (*12*,*14*). Given that 27% of reinfections were asymptomatic, our findings suggest that vaccination might have been protective against infections that workers would have acquired unknowingly. Non-Hispanic Black participants had increased risk for reinfection, which underscores the need for addressing health disparities, especially because racial and ethnic minority groups are overrepresented among essential and frontline workers (*15*).

The first limitation of this study is that sparse data contributed from certain geographic sites and demographic groups reduced the precision of estimates. Second, it is possible that some persons who were reinfected during the early phases of Omicron predominance (when Omicron and Delta were co-circulating) and who also had high Ct values that precluded whole-genome sequencing were misclassified as Omicron reinfections (instead of Delta), although that number is probably negligible, given the rapid acceleration of Omicron dominance. Third, although inclusion of self-reported mask use in community settings was a strength of the study, we were unable to account for venue-specific mask mandates, which might have been variable among participants or changed throughout the study period. Fourth, findings suggesting the need for second and third doses of mRNA vaccines are less relevant as recommendations for additional and variant-specific boosters emerge. Nonetheless, those findings suggest the role of time since immune-modifying events, including most recent mRNA vaccination or previous infection, in reducing risk for reinfection. Overall, our findings underscore the need for maintaining a multipronged approach, including vaccines, nonpharmaceutical interventions, and efforts to reduce disparities, to protect frontline workers as the pandemic enters a period in which reinfections are increasingly common.
